# Abnormal repolarisation after a game of Jass

**DOI:** 10.1007/s12471-023-01844-6

**Published:** 2024-01-16

**Authors:** Hilde E. Groot, Jan A. Krikken

**Affiliations:** Department of Cardiology, University Medical Centre Groningen, University of Groningen, Hanzeplein 1, 9713 GZ Groningen, The Netherlands

## Answer

### Arterial pulse tapping artifact

**Fig. 1:**
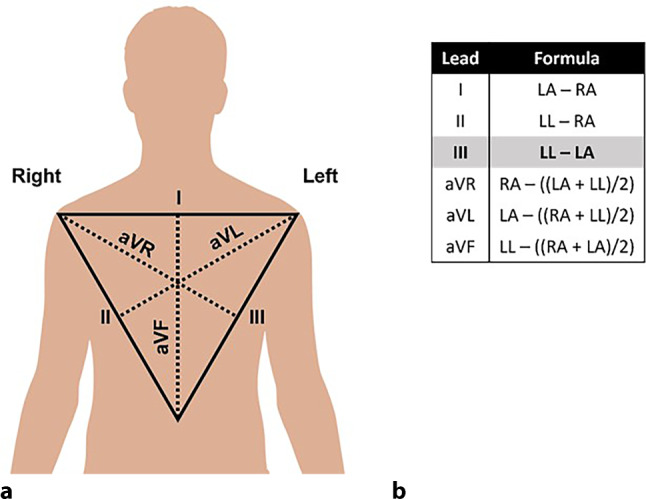
Einthoven’s triangle (**a**). Calculations of lead voltages (**b**). (*LA* left arm, *RA* right arm, *LL* left leg)

The bizarre repolarisation in all leads except lead III is the result of placement of the right arm electrode close to an artery. The artifact on the electrocardiogram (ECG) is known as *arterial pulse artifact* and is caused by movement of the electrode with each pulsation of the artery [1, 2]. Lead III is not affected because this lead does not include the right arm electrode for its voltage calculation (Fig. 1a, b).

The precordial leads are also affected because their average potential across the body is based on the average of the 3 limb leads. The right arm electrode will therefore still influence the precordial lead voltage. This is known as the Wilson central terminal [1].

**Fig. 2:**
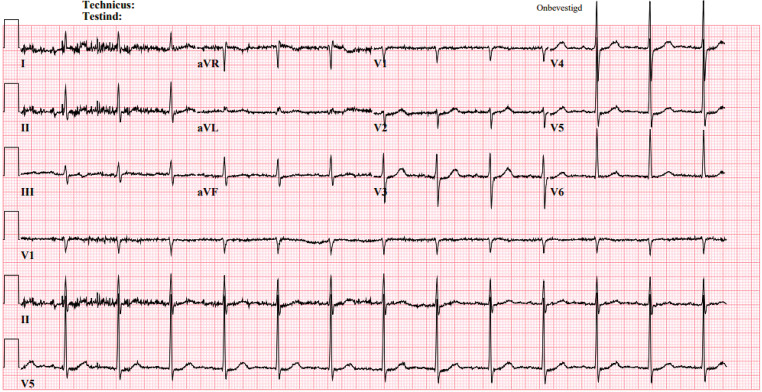
Abnormal repolarisation is absent after positioning the ECG leads

The patient presented no symptoms upon arrival at the hospital. The abnormal repolarisation was absent in the follow-up ECG made at the emergency department (Fig. 2).

Abnormal repolarisation, which is not congruent with a specific distribution, should urge one to carefully inspect patient, ECG, and positioning of the ECG leads. Abnormal repolarisation in all leads except one might be caused by arterial pulse tapping artifact.
